# Stress and Heart in Remodeling Process: Multiple Stressors at the Same Time Kill

**DOI:** 10.3390/jcm13092597

**Published:** 2024-04-28

**Authors:** Fatih Yalçin, Maria Roselle Abraham, Mario J. Garcia

**Affiliations:** 1 Department of Cardiology, UCSF HEALTH, School of Medicine, Cardiac Imaging, San Francisco, CA 94143, USA; roselle.abraham@ucsf.edu; 2 Department of Medicine, University of California at San Francisco, Cardiology UCSF Health, 505 Parnassus Avenue, Rm M314AUCSF, P.O. Box 0214, San Francisco, CA 94117, USA; 3 Department of Cardiology, Montefiore Medical Center, Albert Einstein College of Medicine, Bronx, NY 10461, USA; mariogar@montefiore.org

**Keywords:** hypertension, exercise hypertension, myocardial remodeling, autonomic nervous system, emotional stress, stress score, basal septal hypertrophy, stressed heart morphology, acute stress cardiomyopathy, heart failure

## Abstract

Myocardial remodeling is developed by increased stress in acute or chronic pathophysiologies. Stressed heart morphology (SHM) is a new description representing basal septal hypertrophy (BSH) caused by emotional stress and chronic stress due to increased afterload in hypertension. Acute stress cardiomyopathy (ASC) and hypertension could be together in clinical practice. Therefore, there are some geometric and functional aspects regarding this specific location, septal base under acute and chronic stress stimuli. The findings by our and the other research groups support that hypertension-mediated myocardial involvement could be pre-existed in ASC cases. Beyond a frequently seen predominant base, hyperkinetic tissue response is detected in both hypertension and ASC. Furthermore, hypertension is the responsible factor in recurrent ASC. The most supportive prospective finding is BSH in which a hypercontractile base takes a longer time to exist morphologically than an acutely developed syndrome under both physiologic exercise and pressure overload by transaortic binding in small animals using microimaging. However, cardiac decompensation with apical ballooning could mask the possible underlying hypertensive disease. In fact, enough time for the assessment of previous hypertension history or segmental analysis could not be provided in an emergency unit, since ASC is accepted as an acute coronary syndrome during an acute episode. Additional supportive findings for SHM are increased stress scores in hypertensive BSH and the existence of similar tissue aspects in excessive sympathetic overdrive like pheochromocytoma which could result in both hypertensive disease and ASC. Exercise hypertension as the typical form of blood pressure variability is the sum of physiologic exercise and pathologic increased blood pressure and results in increased mortality. Hypertension is not rare in patients with a high stress score and leads to repetitive attacks in ASC supporting the important role of an emotional component as well as the potential danger due to multiple stressors at the same time. In the current review, the impact of multiple stressors on segmental or global myocardial remodeling and the hazardous potential of multiple stressors at the same time are discussed. As a result, incidentally determined segmental remodeling could be recalled in patients with multiple stressors and contribute to the early and combined management of both hypertension and chronic stress in the prevention of global remodeling and heart failure.

## 1. Introduction

In the cardiovascular disease continuum, short-term or long-term stress induction could possibly result in a variety of pathophysiological scenarios and clinical presentations. Although the types of stressors may be different, myocardial tissue may have some specific morphologic and functional aspects [1,2,3]. It has been pointed out that acute myocardial infarction results in earlier left ventricular (LV) dysfunction compared to chronic hypertension and normal aging [4]. However, we proposed a new paradigm suggesting that certain acute clinical presentations may develop on a chronic pathophysiological base [5,6]. Documentation of a pre-existing chronic base in hypertension-mediated pathophysiology in acutely developed stress-related heart diseases such as ASC could be difficult [6]. Early septal LV remodeling, namely, basal septal hypertrophy (BSH) is related to different stressors like exercise hypertension [1], emotional in acute stress cardiomyopathy (ASC), [2,5,6], functional in hypertension-mediated increased afterload [7], and mechanical in aortic stenosis [8]. We validated BSH as the early imaging biomarker and described stressed heart morphology (SHM) as the specific location for superposed multiple stressors [9].

**Figure 1:**
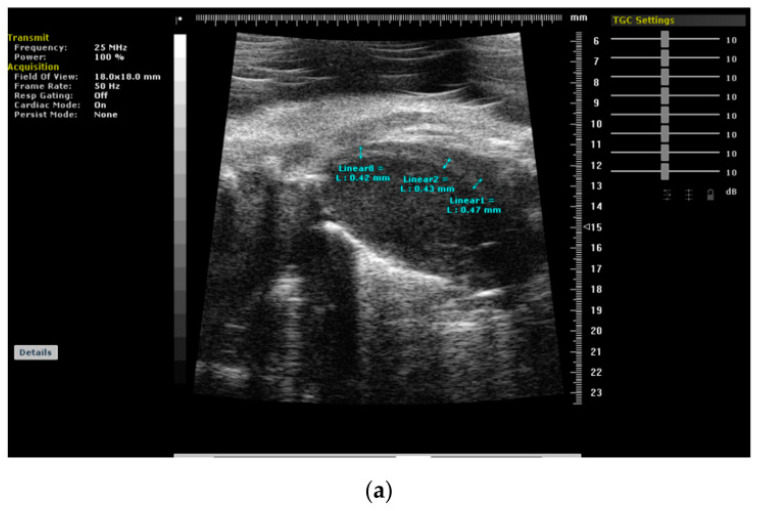

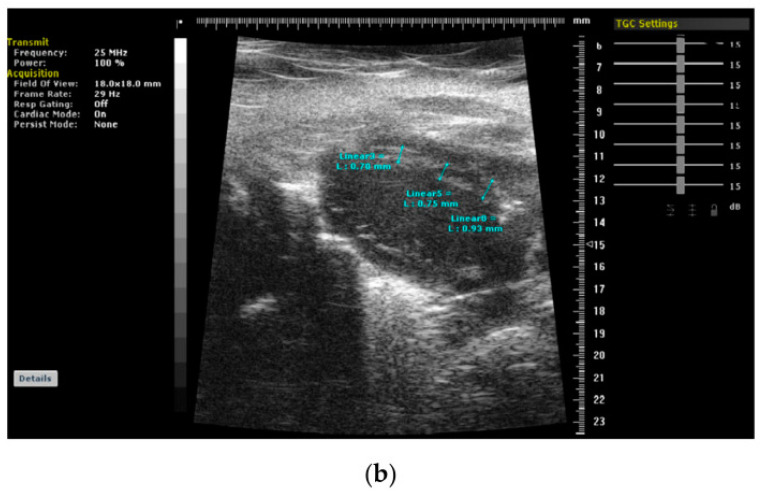
(**a**,**b**) Cardiac images of mice using 3rd-generation microscopic ultrasound show normal cardiac geometry and a regularly remodeled septal wall with thicker septal base at 4 weeks after stress induction due to pressure overload (TAC: transverse aortic construction), respectively.

Since segmental remodeling is not generally used as a method in cardiac imaging, SHM could be underestimated in clinical practice despite it being a mutual finding in both hypertensive heart disease and ASC [5,6,7]. Hypertension is the most common killer in the population, however, approximately half of the total hypertensives stay undiagnosed, according to the World Health Organization [10]. Hypertension in the majority of clinical cases is associated with anxiety, depression, and panic attacks [11]. In our animal validation study, microimaging showed that physiologic and pathologic stressors lead to a very regularly progressed BSH (Figure 1a,b) differently from human beings [12,13,14]. In fact, we have focused on the psychological background in hypertensives with high stress scores who have shown extremely complex, irregular BSH with a myocardial tissue heterogeneity [9,15,16]. This was the main reason for us to describe SHM as the consequence of superposed multiple stressors. Selye began to employ a new term, “stressor” to differentiate between the harmful agent and the biological response more clearly: All agents can act as stressors, producing both stress and specific actions which could lead to some mechanisms including defense or damage in the tissues [17].

Sympathetic innervation is the main factor for cardiac adaptation and LV remodeling under pressure overload and hemodynamic stress [18]. Histological abundance of postganglionic sympathetic neurons is a well-documented explanation for a locally dominant sympathetic overdrive which could be a possible mechanism for the role of a sympathetic drive in BSH [19,20]. The correlation between the autonomous nervous system and hypertension which is an absolute and documented risk factor for recurrent ASC [21] is commonly associated with emotional stress [17].

## 2. Chronic Stress

**Figure 2:**
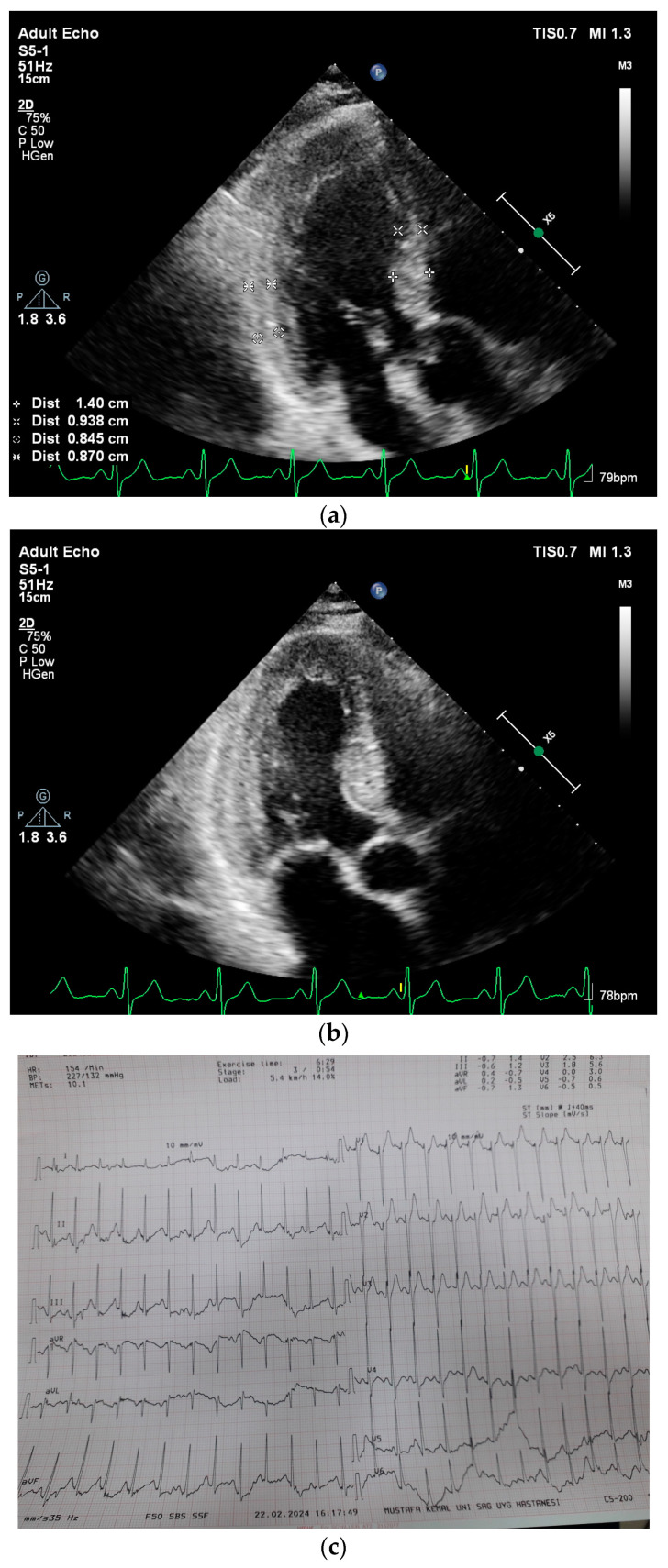
A slight curve of interventricular septal base from apical 4 chamber view during end-diastole (**a**) and end-systole (**b**) in a patient with exercise hypertension (a greater blood pressure response than 210/110 mmHg to peak exercise stress), (**c**) and basal septal hypertrophy.

Stress is associated with both the psychological state and the physiological response of the body which can lead to anxiety and depression, as well as high blood pressure [11]. Therefore, hypertensive subjects could be facing severe emotional problems and develop a predominant septal base prior to acute episodic clinical presentations. In fact, episodic ASC could be an LV dysfunction due to both hypertension [21] and chronic stress as we have recently emphasized [6]. A variety of stressors such as exercise hypertension (Figure 2a–c) could be superposed over the LV septal base which is the unique part of a heart tissue free from parasympathetic modulation [9].

## 3. Stress and Brain

Chronic stress could be more important than we think and preparing a base for the future acute episodic attacks as Dr. Ratey JJ. has pointed out, chronic stress is a threat to the body’s balance and can tear at the architecture of the brain [11]. Additionally, he also emphasizes that emotions may not be separated by biological aspects. Furthermore, he describes that anything which activates the brain’s cellular activity is a form of stress for the brain and the body. It looks difficult to separate emotions and relevant chronic cognitive abnormalities in human beings from blood pressure regulation pathways as well as hypertensive LV remodeling which is basically related to the sympathetic overdrive [5,6,9]. For the brain tissue, it was pointed out that plenty of activity is formulated by body movements which activates the specific neuronal signaling pathways between the body and the brain and these interactions produce emotions [11].

In fact, emotional reactions are associated with the general response of brain cellular activity under stress and could prepare a base with repetitive chronic stress, especially in the individuals with high stress scores prior to the acutely developed stress-induced syndromes [6]. Over the past years, there is increasing evidence about the brain–heart interaction with major potential implications for the treatment of cardiovascular diseases. Cerebrovascular accidents and transient ischemic attacks are frequently caused by hypertension and cardiac arrhythmias [11,22]. Brain architecture could be harmed by heart problems even in the absence of manifest stroke and atrial fibrillation is a risk factor for cognitive impairment and hippocampal atrophy [22,23]. Cognition and measures of structural brain integrity are important in cardiovascular problems. Panic disorders and emotional distress such as ASC may give rise to tachyarrhythmias with ensuing transient LV dysfunction [24]. We recently have pointed out that chronic stress-mediated SHM as a validated early imaging biomarker possibly plays a pre-existing role and prepares a base for acute emotional episodes [5,6,7,9,14,15,16].

## 4. Stress and Heart

We proposed that the complex septal base, SHM, is the specific conjunctive point of determination in a variety of stressors and represents the adaptive phase of LV remodeling [25]. SHM could take a dominant role as the early imaging biomarker in the management of multiple stressors at the same time in the near future. SHM could be more important than we think because it represents not only a specific morphologic aspect but some functional tissue aspects. Interestingly, cardiac response to stress like the brain tissue is related to cellular activity as we have published some clinical reports showing hyperdynamic myocardial response using fluid and tissue dynamics to stress induction [2,26,27,28]. Tissue adaptation independent from the type of stress has been generally accepted as a defense mechanism that uses accumulated energy. However, maladaptation of tissue to chronic stress is possibly related to the terminal phase with diminished cellular activity and accumulated energy which is generally accepted as the tissue damage phase [7,17,25].

Like the relation between a variety of body movements and the emotional response of the brain tissue [11], cardiovascular findings and emotions are also related [15,16,17,21,22,23,24]. While chronic cardiovascular disease progression has a complicated and multifactorial transaction including a genetic aspect, some potential missing links could possibly exist between chronic risk factors and sudden attacks like acute coronary syndrome (half of them may not be explained by classical risk factors) or ASC [1,2,3,5,6,7,14,15,16]. Beyond stress mechanics affected by systemic neurohormonal activation [17,29], sympathetic nerve activity could have an additional role [18,19,20].

## 5. Updated Knowledge for Superposed Multiple Stressors

**Figure 3:**
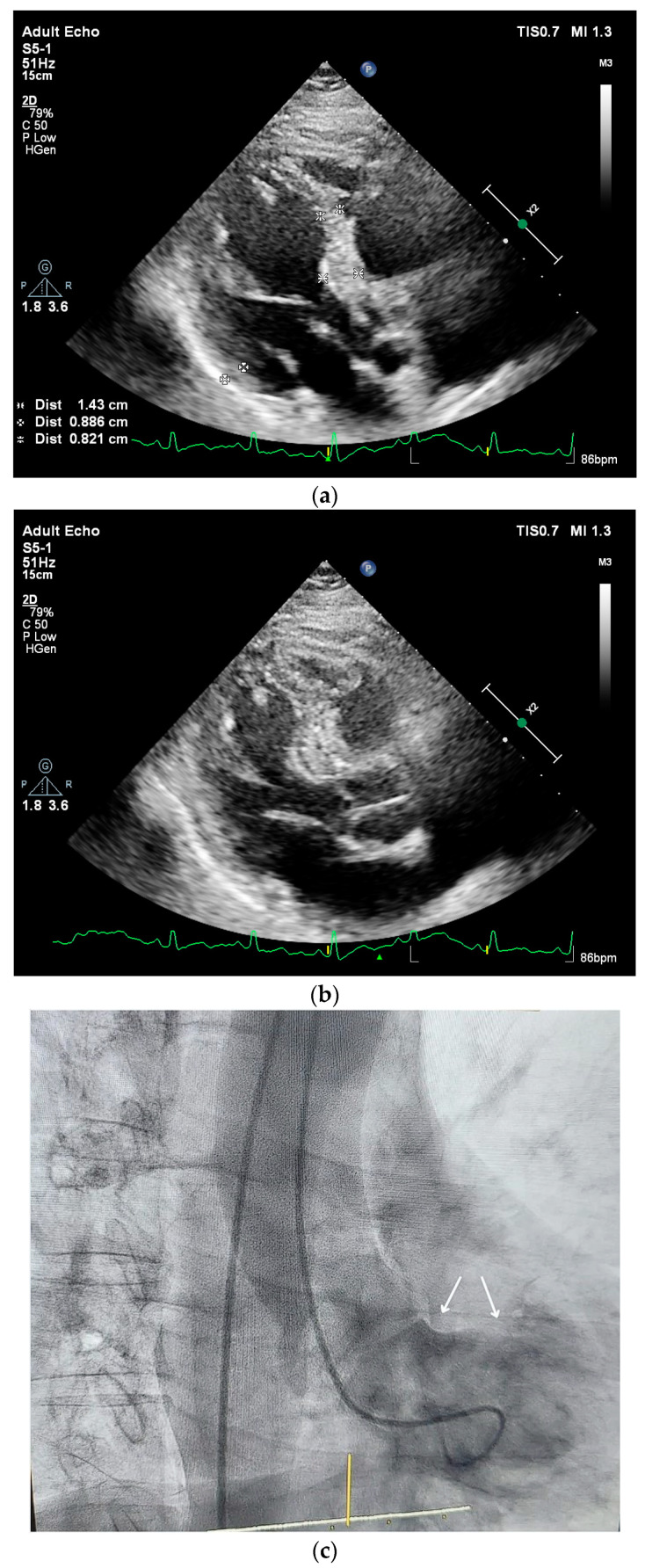
(**a**) Predominantly placed hypertrophy over septal base from apical 4 chamber view during end-diastole in a previously undiagnosed hypertensive patient with SHM who has undergone a recent earthquake and lost his two children during catastrophy. (**b**) A remarkable protruding basal septum with myocardial tissue heterogeneity into the LV cavity during systole in the same patient with a high level of stress score. (**c**) A remarkable basal cavity narrowing (pointed with white arrows), “Tako-tsubo like cardiac geometry” due to basal septal hypertrophy of the same patient in ventriculography.

**Scheme 1:**
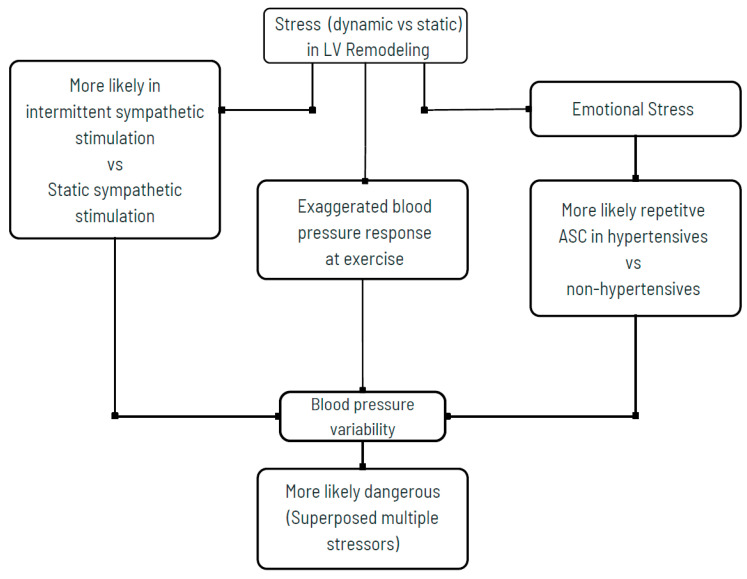
The hazardous effect of superposed multiple stressors.

1. SHM could have some specific geometric and functional aspects affected by a variety of stress stimuli or superposed multiple stressors [9,14,15,16].
2. Segmental remodeling, namely, SHM could be implemented in a clinical protocol for monitoring previously undiagnosed hypertension and increased stress [9,30,31,32].
3. The effect of superposed multiple stressors is independent from the type of stress and complex SHM with tissue heterogeneity in human beings (Figure 3a–c) having a striking difference from the regular segmental remodeling progression in small animals under stress induction determined by microimaging [12,13,14].
4. Segmental LV remodeling instead of cross-sectional measurements could contribute to the prediction of future global LV remodeling and heart failure development due to basal apical discordance as we have recently proposed in patients with mechanic stress in aortic stenosis and functional stress in hypertension due to increased afterload, respectively [25,30,31,32].
5. The dominant role of the type of stress is not known in SHM, since there is a lack of the pooled data regarding segmental remodeling around the world as we recently have pointed out in our editorial [31].
6. There is a need to work on cell biology to search cellular levels of myocardial tissue to prove SHM as the specific location of Selye’s theory on nonspecific general adaptive responses to stressors [17].
7. Importantly, literature data showing that intermittent adrenergic stimulation [33] in animal models is more dangerous than static sympathetic stimulation in terms of LV remodeling development (Scheme 1).
8. We pointed out the importance of stress-induced exaggerated hypertension and hemodynamic overload under stress in the patients with BSH [1,15,25]. Years later, we realized the importance of emotional factors on very complex BSH [2,5,6,7,15,16,31] after we validated BSH as the early imaging biomarker during very regular LV remodeling in small animals using 3rd-generation microscopic ultrasonography [12,13,14].
9. In addition to increased stress scores and more cognitive problems in hypertensives [6,15,16] compared to non-hypertensives, it was shown that recurrent ASC is more commonly detected in hypertensive patients possibly due to hemodynamic fluctuations [21,34]. Long-term variability in blood pressure is now known to be related to increased mortality than the effect of mean blood pressure [35,36].
10. In addition to its relation to SHM, superposed multiple stressors including increased adrenergic overdrive [9,21,34], cognitive disorders [6,15,16], chronic or exercise hypertension [1], which are also associated with increased cardiovascular mortality [37,38], are more likely dangerous since each risk is associated with LV remodeling with hemodynamic overload [1,2,5,6,7,8,9,25,26,27,28] and represents hemodynamic fluctuations due to blood pressure variability which is associated with increased mortality [35,36].

## 6. Future Perspective

Hypertension could possibly be the case prior to the first ASC episode since recurrent ASC attacks relate to hypertension. Combined daily stressful components like exercise hypertension, emotional stress, or excessive sympathetic overdrive like pheochromocytoma result in hemodynamic overload with blood pressure variability that could be a potential killer like exercise hypertension and prevented by comprehensive neuro-cardiologic perspective. SHM should be recorded globally and evaluated more comprehensively since all stressful components could potentially be determined in SHM patients.

**Figure 4:**
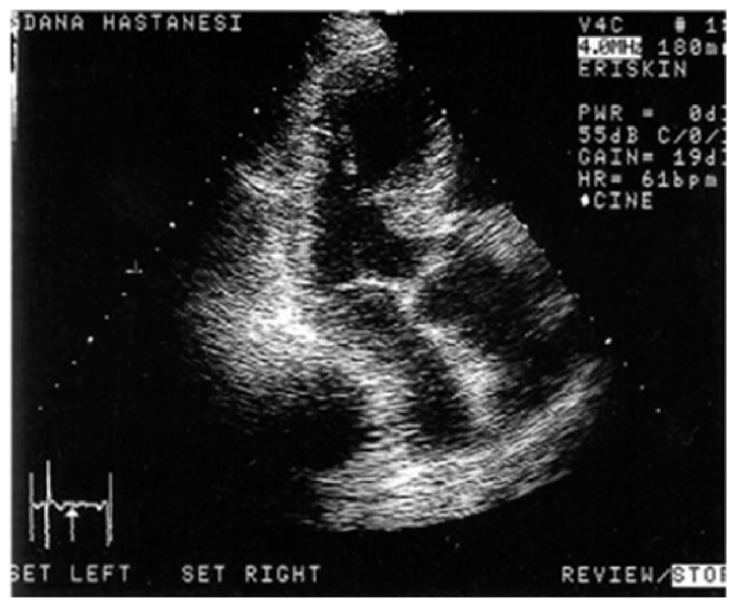
Enormously localized SHM over septal base in a hypertensive patient, a parliament representative with very high stress score [5,15].

A variety of stressors could be superposed over the septal base as the specific location which is the unique human tissue free of parasympathetic modulation. Anxiety, depression, and panic attacks are not rare in hypertension. Since undiagnosed hypertensive heart disease has become more prevalent, we strongly suggest segmental evaluation instead of single cross-section and point out the importance and potential advantages of determination of echocardiographic segmental geometric data. Beyond pure hemodynamic overload, SHM does not spread over other myocardial segments (Figure 4) which is why combined assessment of the heart and brain will possibly have an additional advantage to preventing “the hazardous effect of superposed multiple stressors” (Scheme 1).

Moreover, this new paradigm could be beneficial to explore missed cases with hypertension and to document whether or not these individuals have increased stress score prior to acute stress-related cardiac attacks. Sympathetic overdrive with palpitation, sweating, tremor, red face, etc., in pheochromocytoma could be the most typical presentation of superposed multiple stressors including hypertension and emotional stress. Episodic emotion-mediated hemodynamic fluctuation with blood pressure variability due to multiple stressors at the same time in humans is a consistent finding with the hazardous effect of intermittent sympathetic stimulation in animals.

Baroreflex sensitivity and heart rate variability are important parameters in the unique role of the autonomic nervous system on both the heart and brain. In fact, we detected that respiratory exercise has a special role in clinical practice which can provide an important benefit in the control of combined hypertension and emotional problems [39]. In addition to classic prevention using salt restriction and drugs, activity vagal stimulation, exercise training with blood pressure control, electrical neurostimulation, music therapy, and, recently, global assessment and management of hypertensive patients with high stress scores have become interesting topics.

## 7. Stress History

Adaptation to stressors independent from the type of stress is related to a systemic neurohormonal mechanism, namely, general adaptation syndrome [40]. Clinical features of disease could be the result of a failure in the nonspecific adaptive mechanisms of the body [41]. General adaptation syndrome was described by Selye and adrenal glands were shown as the critical organ that plays a central role in adaptive reactions [42]. Acute and prolonged overstimulation by physical or emotional trauma affects the body leading to the release of adrenaline and having a harmful effect on the previously balanced system in terms of function and geometry, as well as energetics [43].

Selye introduced a new term as stressor which describes both harmful agent and the biological response, therefore, all agents can act as stressors, producing both stress and specific actions [5]. Since there is difficulty in differentiating the harmful agent and the biological response, high technologic methods were used longstandingly, but certain separative formulas could not have become clinically practical. Selye also used a new notion as adaptation energy [43] in description of sufficient tolerance to different forms of injury, showing the stage of tissue resistance. He also mentioned the limited energy as eventual exhaustion and death due to repeated environmental stress [41]. Stress also was interpreted as the interaction between damage and defense or force and resistance which is completely similar to adaptive response to stress before tissue damage [17]. Stress is used practically as the common denominator of all adaptive reactions in the body and more specifically, stress, rather than adaptation, indicated the central biological course at the general and complex reactions in the body [17]. Stress acts not only as an external trigger of internal processes but as the physiological or pathological process itself [40].

Selye’s attempt to develop a novel theoretical framework for understanding a range of biological reactions and clinical manifestations built up a base at the endocrinologic level, which is basically a neurohumoral response to external trauma describing nonspecific general adaptive response without any struggle to describe a specific location, function, or mechanism beyond a systemic response due to neurohumoral activation in circulation. Nevertheless, stress-related studies persist until much more experimental work has been conducted to separate specific physiological effects from those of a nonspecific nature [44]. Selye’s studies of adaptation and stress were supported financially and the general adaptation syndrome constituted a philosophical point of view for disease concept. Those works were also evaluated by medical platforms and resulted in a new data pool of Bethesda regarding the new disease concept [45].

## 8. Autonomic Nervous System and Stressed Heart Findings

Neurologic perspective and modern neuroendocrinology were partly built up by the intellectual depth and remarkable energy in Selye’s work on contemporary biology [46]. Stability in mental and emotional processes is possibly provided by the interactions of the body’s subsystem interactions which needs a synchronization between the two branches of the autonomic nervous system (ANS) [47]. Sympathetic and parasympathetic activity in ANS with heart–brain synchronization contribute to the stability of the processes to maintain cardiovascular health with the regulation of heart rate, blood pressure at rest, and under physiologic and pathologic stress.

Balance of ANS is an important target to regulate for cardiovascular stability including the resetting of baroreceptor sensitivity, which is related to the improvement in short-term blood pressure control and increased respiratory efficiency. ANS balance also provides increased vagal afferent traffic, which is placed in the function of inhibiting pain signals and sympathetic outflow. In addition, ANS balance will contribute to the control of increased cardiac output with increased ability of the cardiovascular system to adapt to circulatory requirements [47]. After the importance of ANS in cardiovascular problems was understood clearly, new efforts and perspectives regarding increased cardiovascular risk factors like hypertension with emotional problems were started [39]. These efforts focus on the interrelation of multiple stressors different from the scientific efforts of Selye on the separation of physiological or pathological processes as the internal responses to external triggers or stressors [9].

In cardiovascular diseases, ASC, which is a clinical spectrum including emotional stress and related temporary LV dysfunction, seems to be the most typical phenomenon started by emotional instability [5]. In patients with ASC, an increased wall stress of acute emotional stress on the midapical part as the pathophysiologic mechanism leads to an episodic decompensation [6]. The regional LV base is relatively more resistant to stress compared to the midapical region in this phenomenon and is associated with a hyperdynamic aspect under emotional stress [5]. Similarly, hyperdynamic basal tissue with focal basal hypertrophy is also the case in hypertensive patients [26]. In the chronic period, episodic stress induces the heart to give a response of high heart rate-pressure product and high LV outflow tract blood flow, as well as basal septal hyperdynamic tissue in BSH patients with hypertension [27].

We described SHM because the predominant LV base with adaptive hypercontractility and a relatively larger midapical cavity is a conjunctive point of determination in clinical conditions, not only with chronic stress due to increased afterload in hypertension but with acute emotional stress in ASC [2,5]. These clinic observations possibly represented a compensatory segmental adaptation to increased stress stimuli in this group of patients. Beyond these cross-sectional observations, we decided to determine the progression of LV segmental remodeling prospectively. We used treadmill exercise for physiologic and transaortic construction for pathologic stress stimuli and 3rd-generation microscopic ultrasonography in the small animals for the determination of segmental LV remodeling evolution. In this study, we noted for the first time that BSH is the early imaging biomarker of LV remodeling under both physiologic and pathologic stress [12,13]. Nevertheless, since we detected BSH development as the initial LV remodeling prospectively in animal models in both physiologic stress-mediated remodeling with normal organization of myocytes and pathologic with myocyte hypertrophy and collagen accumulation, correct diagnosis for the nature of human BSH should be documented by blood pressure monitorization to explore whether or not pressure overload is the case in clinical practice [1,2,3].

We believe that these segmental aspects of LV under stress are important in a variety of clinical conditions with acute or chronic stress [5,6,7]. Animal validation studies years later than our description of SHM provided an understanding of the effect of high stress scores on the complex morphology over BSH in human beings differently from regular segmental remodeling in animal models under pathologic stress due to pressure overload [14,15,16]. Beyond emotional etiology, we mention the morphologic importance of segmental LV remodeling in stress-related cardiovascular diseases including mechanical stress due to aortic stenosis which is associated with SHM [8,32].

## 9. Nonspecific Stress Adaptation of Selye and Segmental Remodeling

Furthermore, it could be extremely difficult to find the dominant type of stress-shaping complex SHM in humans with exercise hypertension which has combined physiologic and pathologic stress. Except for the emotional component, both pressure overload as pathologic and treadmill exercise as physiologic stress in animals represent human exercise hypertension in the real world [14,15,16]. In this new paradigm, a complex heart base signifies the importance of cognitive function in humans [9]. Hypersensitivity of this special tissue to multiple stressors which could be superposed over the heart base could be a substrate for further research to explore whether this finding has a specific role in the adaptive process under nonspecific external stressors which are independent of acute, chronic, or focal neurohumoral response in myocardial tissue or systemic neurohumoral response as hypothesized by Hans Selye [17,40,41,42,43].

While blood pressure variability or exaggerated blood pressure response to exercise is important cardiovascular risks, improvement in both emotional and hemodynamic balance with blood pressure stability can contribute to cardiovascular health. This mode is associated with reduced perceptions of stress, sustained positive affect, and a high degree of mental clarity and emotional stability. We noted in hypertensives that specific rhythmic breathing methods may induce heart rhythm coherence and stability of increased blood pressure and depression [39].

In the respiratory–sympathetic connection with hypertension, the respiratory and circulatory systems are both involved in the delivery of oxygen and removal of carbon dioxide in the tissues [48]. Any alteration in this connection could result in cardiovascular consequences. Indeed, it was observed that this connection could lead to an increase in respiratory modulation of sympathetic overdrive that possibly contributes to the development of hypertension [49]. Both healthy and active people can experience a variety of limitations due to weak respiratory muscles. An increased respiratory–sympathetic connection may be responsible for the difficulty in the management of hypertension in humans [48,49].

In fact, studies have demonstrated that the strength of the inspiratory muscle plays a crucial role in the pathophysiology of exercise limitation in a variety of clinical disorders [50]. Inspiratory muscle weakness was detected in hypertension, one of the increasingly common risk factors and exercise capacity was negatively affected by this weakness [51]. Research studies on respiratory exercises that effects exercise capacity, one of the alternative treatments of hypertension, recently have been gaining importance. Ublosakka-Jones et al. [52] found that respiratory exercise training given at a low load for 8 weeks was effective on arm endurance capacity. An improvement in respiratory performance capacity caused by muscle endurance training in normotensive elderly patients continued with prolonged active training for 5 weeks [53]. It was observed that an 8-week workload applied to the inspiratory muscles can increase exercise capacity [54]. It is known that individual quality of life with hypertension is worse than normotensive individuals [55].

Breathing control is beneficial in lowering blood pressure in hypertension patients [56]. Respiratory exercises are beneficial due to ANS which impacts physiological respiration, emotion, and cognition mechanisms [57]. These exercises reduce sympathetic nervous system activity while enhancing parasympathetic nervous system activity, linked to cardiac vagal tone. Consequently, these exercises influence emotions, emotional regulation, psychological adaptation, reactivity, expression, and empathic responses. Utilizing this mechanism, breathing exercises can potentially be beneficial for conditions like depression [57].

We observed that device-assisted respiratory exercises performed with or without a certain workload yielded a benefit in the severity of depression in hypertension patients [40]. In our study examining the effect of inspiratory muscle training on quality of life in patients with hypertension, we observed that 8 weeks of breathing exercises training, including whether unloaded, low load intensity, or high load intensity training, decreased resting blood pressure in hypertension patients [39]. Breathing exercises and cardiovascular modulation work are crucial components of maintaining cardiovascular homeostasis and respiratory training is an effective method in the effective control of blood pressure [58].

Evaluation of hypertensives with a combined antihypertensive approach and stress management under a neurocardiologic perspective with comprehensive diagnostic tests are unmet needs in current clinical practice. We noted that incidentally determined segmental LV remodeling and basal septal hypertrophy using basal and midapical measurements are crucial, instead of a single cross-sectional measurement on the cardiovascular imaging. Specific tissue aspects of SHM could be implemented in a practical clinical protocol not only for previously undiagnosed hypertension but cognitive disorders in clinical practice.

SHM should also be implemented in a practical clinical protocol for monitoring future acute episodes due to hypertension. Evaluation of hypertensives with a combined antihypertensive approach and stress management under a new neurocardiologic perspective with comprehensive diagnostic tests are currently unmet needs. In conclusion, incidentally determined segmental LV remodeling, namely, SHM, could be recorded during clinical practice globally and evaluated more comprehensively [9]. Beyond the clinical aspect of SHM, there is a need to focus on cell biology to work on cellular levels of myocardial tissue to search whether SHM is the specific location of Selye’s nonspecific general adaptive response to stressors.

## Abbreviations

SHM	stressed heart morphology
BSH	basal septal hypertrophy
ASC	acute stress cardiomyopathy
LV	left ventricular
ANS	autonomic nervous system
